# A Workshop to Reflect on Personal Resilience in Emergency Medicine Residents: Applying the Connor-Davidson Resilience Scale, Visual Explorer, and the Critical Incident Questionnaire to Support Introspection

**DOI:** 10.7759/cureus.8597

**Published:** 2020-06-13

**Authors:** Alanna O'Connell, Mansoor Siddiqui, Anthony Sielicki, Robin Naples, Dimitrios Papanagnou

**Affiliations:** 1 Emergency Medicine, Thomas Jefferson University, Philadelphia, USA; 2 Emergency Medicine, Henry Ford Hospital, Detroit, USA; 3 Emergency Medicine, Thomas Jefferson University Hospitals, Philadelphia, USA

**Keywords:** resident wellness, wellness, resilience, emergency medicine resident, resident resilience

## Abstract

Burnout among emergency medicine (EM) residents is gaining increasing attention. The authors designed a workshop to assess EM residents’ resilience using a validated scale to prompt personal reflection. The workshop then shifted to peer-to-peer conversations and sharing using images from Visual Explorer (VE) to further reflect on resilience. Overall, resident resilience scores were below those of the US general population, with post-graduate year (PGY)-2 year residents having the lowest scores. The workshop was well received by residents; data from the Critical Incident Questionnaire (CIQ) suggested that residents felt engaged during discussion of the images. Further study is needed to assess the correlation between resilience scores and burnout.

## Introduction

Resident burnout is gaining increasing attention as an important topic in graduate medical education (GME). Burnout is characterized by poor self-care, dehumanization, exhaustion, and reduced effectiveness. Due to recent tragedies of physician suicide and an increased awareness of the impact burnout, the study of wellness and resilience among EM providers and trainees has recently blossomed. Even though there are ongoing discussions on practices to best address burnout, methods have focused on promoting resilience, mindfulness, and provider engagement [1].

The Accreditation Council for Graduate Medical Education (ACGME) has recommended including wellness into resident curriculum but it is often difficult to integrate time for reflection and discourse on provider personal wellness into formal programming due to competing curricular demands. In these limitations, we sought to develop a meaningful workshop that would allow EM residents the opportunity to reflect on their resilience and create a ‘space’ that would allow them to share outlets that serve to recharge them amidst their training.

Several instruments have been developed to help describe wellness patterns, engagement, as well as resilience. One of these is the Connor-Davidson Resilience Scale (CD-RISC, http://www.connordavidson-resiliencescale.com) which is a psychometrically valid, reliable, and commercially available scale developed to objectively measure and monitor resilience in patients diagnosed with post-traumatic stress disorder (PTSD). It provides a quantitative score of resilience and also offers normative scores for the US general population. For our workshop, the CD-RISC was purchased and used as the instrument for residents to reflect resilience.

The workshop is informed by the work of Heron and Reason [2], and is based on the concept of presentational knowing. Heron and Reason introduced four ‘ways of knowing’ that are defined both in terms of process and outcome. The concept of presentational knowing refers to knowledge that an individual creates and communicates through expressive imagery in lieu of language, which may often constrain discovery and knowledge. This may include movement, dance, sound, music, drawing, painting, sculpture, story, and images. By having residents answer the framing question (i.e., “What makes you resilient?”) through imagery (i.e., selecting a single image from a series of random images), they had the opportunity to tap into a deeper understanding of what makes them resilient, and, in the process, potentially reveal unconscious assumptions they never previously critically thought about. 

Through this workshop, we aimed to study the differences in CD-RISC scores in and across resident classes at our institution and determine how they compared to the general US population. Our secondary objective was to determine if such a workshop is meaningful in promoting self reflection. Given the high incidence of burnout among EM physicians, we hypothesized that the resident scores would be lower than the general US population.

## Technical report

Design considerations and education context

The workshop was designed for all post-graduate year (PGY) levels in EM at Thomas Jefferson University, a three-year EM training program located in Philadelphia, PA.

Design protocol and development

We used the CD-RISC instrument for residents to self-assess their resilience for the workshop. We then used the Visual Explorer (VE) set of images to guide discussion and personal reflections.

The workshop was scheduled for 90 minutes during the regularly scheduled, once weekly EM resident conference. A 10-minute opening presentation was given by the facilitator who introduced the workshop, described the importance of resilience, and discussed the CD-RISC tool. Residents were then given 10 minutes to complete the 25-item CD-RISC instrument. The scores for the general population were provided to them, which allowed them to see how they numerically compared with the general public. The residents were divided into small groups and were given the optional opportunity to share their reactions to their scores within their small group peer groups.

Following CD-RISC self-assessment, the facilitator then introduced the VE exercise and its instructions. VE is a set of over 200 evocative images created and sold by the Center for Creative Leadership [3]. VE has been successfully used in organizations to promote reflection, collaboration, and creative conversations among participants. Facilitators of the VE exercise typically provide participants with a framing question that helps guide their selection of an image that best captures their personal reflections. Prior to the beginning of the workshop, >200 VE images were placed on tables in a separate room that adjoined the conference room. They were instructed to first reflect on the framing question, “What makes you resilient?” After reflecting on this question for five minutes, residents were asked to move to the next room, to browse through all the images, and to choose the single image that best resonated with their respective reflections. Residents were given 10-12 minutes to complete this task.

After all residents chose their images, they were asked to sit down and reflect on their image of choice. Specifically, they were asked to answer five reflective questions. Questions can be seen in Table 1 below.

**Table 1:** Questions given to residents to help them reflect on their image.

Question number	Reflective question
1	What is the image that you chose?
2	What is happening in the image?
3	What is the context of the image?
4	Is there anything surprising about the image you chose?
5	How does the image connect with the framing question about what makes you resilient?

Residents were given 10 minutes to answer these questions.

Within their small groups, residents were asked to take turns to present their images and share how their selected images tied to the framing question on what makes them resilient. Residents who listened to the presenter were then asked to share their observations of the image, and share subtle findings that may not have been described by the presenter. Each resident in the group was given the opportunity to present her/his image. The remainder of the workshop was allocated for this segment (30 minutes).

Evaluation methods

Prior to closing the workshop, participant volunteers were asked to share any surprises and/or insights gained through the dialogue with their peers. The facilitator then closed the workshop, and asked residents to complete a brief survey.

Participants completed a brief five-item survey based off of Stephen Brookfield’s Critical Incident Questionnaire (CIQ) [4]. The questions and responses can be seen in Table 2 in the results section below.

**Table 2:** Brookfield questionnaire responses with the most frequent responses and the response rates.

Question	Most common response	Response recurrence
At what moment during the workshop did you feel most engaged with what was happening?	Discussing photos	11 responses
At what moment during the workshop did you feel most distanced from what was happening?	During lecture	9 responses
What actions did anyone take (facilitator or peer) that you found most supportive or helpful?	Peers sharing what makes them resilient	6 responses
At what point in the workshop did you feel most confused?	At no point	7 responses
What surprised you the most during the workshop?	Peer's image choice and it what meant to them	4 responses

Evaluation results

Thirty residents participated in the exercise with one primary facilitator and two additional faculty members to help facilitate small group discussions. There were eight PGY-1 residents, nine PGY-2 residents, and eight PGY-3 residents who participated. This was a total of 25 residents and survey responses.

National data from the Connor-Davidson Score Interpretation guide states that the US general population mean is 80.4 [5]. They arrange the scores into quartiles. The lowest quartile placed the bottom 1%-25% of the US population into score ranges from 0 to 73. The second quartile placed 26%-50% of the population from scores 74 to 82. The third quartile placed 51%-75% of the population with score ranges from 83 to 90. Lastly the fourth and top quartile placed 76%-100% of the population into scores from 91 to 100. 

**Figure 1:**
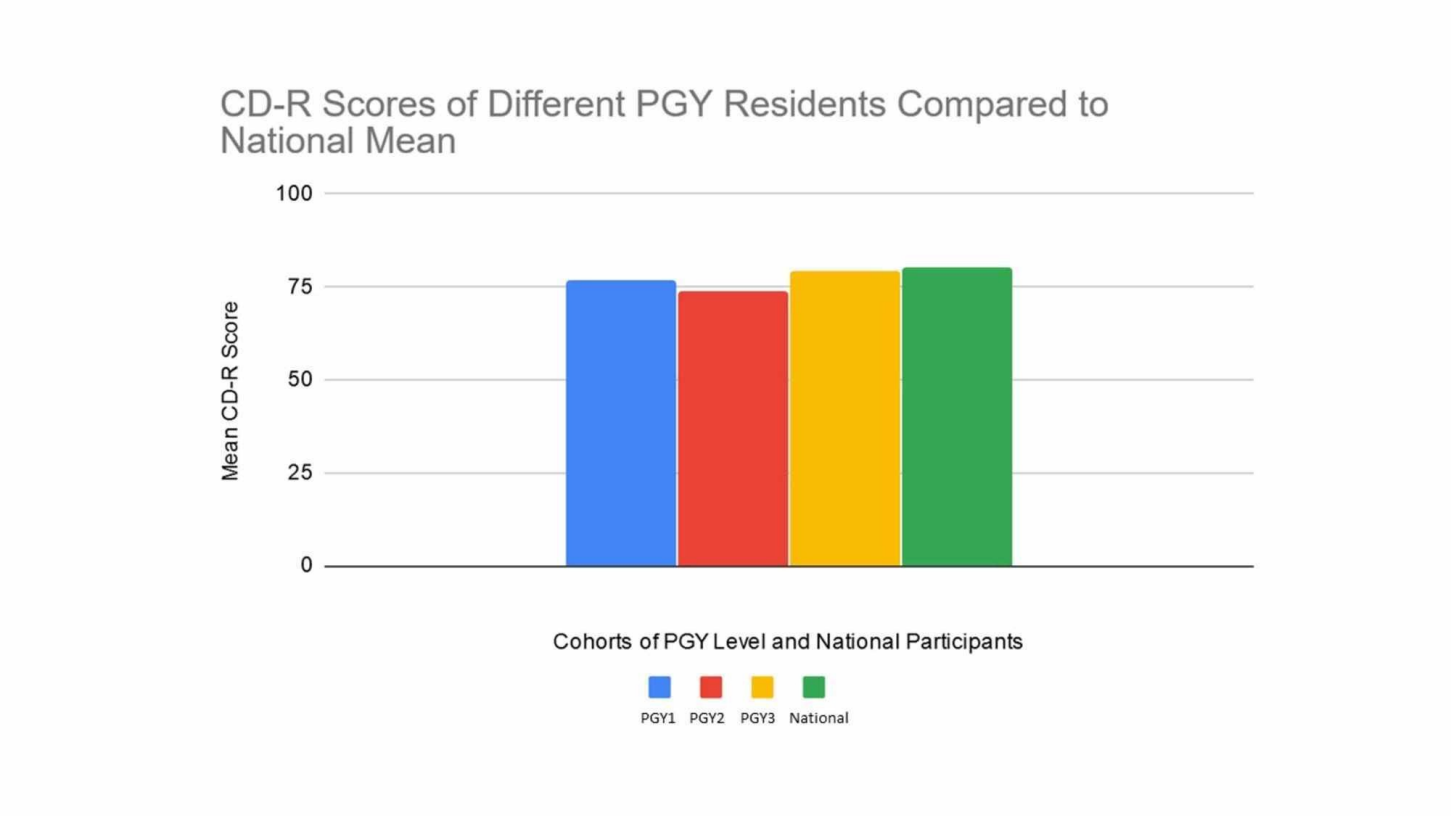
CD-R scores of residents participating compared to national mean.

 Our resident’s overall mean score was 76.5. This placed them in the second quartile for the US general population. The eight PGY-1 residents had a mean score of 77; The nine PGY-2 residents had a mean score of 73.8; and the eight PGY-3 residents had a mean score of 79.3 (see Figure 1 below).

From the Brookfield questionnaire, the most common responses can be seen in Table 2 below.

The point in the workshop where residents felt most engaged was during discussion of photos (11 responses). The point in the workshop where residents felt most disengaged was during the lecture (nine responses). The action the facilitator took which was found to be most helpful was having peers share what makes them feel resilient (six responses). The point in the workshop where participants felt most confused was “at no point” (seven responses). Finally the question “what surprised you the most during the workshop” had a most common response of: peer’s image choice and what it meant to them (four responses). 

## Discussion

One of the most striking findings in our data was the comparison of mean scores from the residents to the national average. As hypothesized, our residents in this study had lower resilience scores compared to the general US population. While this study did not study the level of burnout amongst our residents, future studies should examine the correlation between resilience and burnout. 

An interesting finding in our data pertained to PGY-specific trends in resilience scores. The PGY-2 year had the lowest average CD-R scores at 73.8%. This is an interesting finding as previous studies had found that burnout tends to be especially prevalent in the early years of training, particularly in the transition from end of PGY-1 year into PGY-2 [6] . Another study had found that PGY-2 residents had lowest scores on an Emotional Quotient Inventory (EQ-i) compared to their peers [7]. If their resilience score being low was consistent with a level of burnout this would be consistent with those studies. The highest mean scores were observed among PGY-3 residents at (79.3%), which was at the national average.

 Unfortunately our study was limited by the small number of participants. Further research may include residents at other programs to more robustly study the level of resilience among US EM residents, with correlation studies to determine a linkage burnout. 

## Conclusions

Given the increasing focus on burnout in medical professionals, the authors examined this problem for the lens of resilience in a workshop format. Residents found the workshop to be helpful, especially during the discussion and sharing of visual images to support introspection. Further study is needed to better understand the role resilience plays in burnout, and do determine if trends exist in the development of resilience across residency training. 
